# Anthropometric, clinical and molecular determinants of treatment outcomes in postmenopausal, hormone receptor positive metastatic breast cancer patients treated with fulvestrant: Results from a real word setting

**DOI:** 10.18632/oncotarget.16982

**Published:** 2017-04-09

**Authors:** Laura Pizzuti, Clara Natoli, Teresa Gamucci, Mariella Mauri, Domenico Sergi, Luigi Di Lauro, Giancarlo Paoletti, Enzo Ruggeri, Laura Iezzi, Isabella Sperduti, Lucia Mentuccia, Agnese Fabbri, Marcello Maugeri-Saccà, Luca Moscetti, Maddalena Barba, Patrizia Vici

**Affiliations:** ^1^ Division of Medical Oncology 2, Regina Elena National Cancer Institute, Rome, Italy; ^2^ Department of Medical, Oral and Biotechnological Sciences, Centro Scienzedell’ Invecchiamento e Medicina Traslazionale-CeSI-MeT, Chieti, Italy; ^3^ Medical Oncology Unit, SS Trinità Hospital, Loc. San Marciano, Sora, Frosinone, Italy; ^4^ Division of Oncology, San Giovanni Hospital, Rome, Italy; ^5^ Division of Oncology, Complesso Ospedaliero Belcolle, AUSL Viterbo, Strada S. Martinese, Viterbo, Italy; ^6^ Biostatistics Unit, Regina Elena National Cancer Institute, Rome, Italy; ^7^ Scientific Direction, Regina Elena National Cancer Institute, Rome, Italy; ^8^ Department of Medical and Surgical Sciences for Children and Adults, Azienda Ospedaliero-Universitaria Policlinico di Modena, Modena, Italy

**Keywords:** fulvestrant, hormone receptor positive metastatic breast cancer, endocrine sensitivity, endocrine resistance

## Abstract

To characterize determinants of treatment outcome in a real world population of 161 post-menopausal hormone receptor-positive metastatic breast cancer patients treated with fulvestrant. Descriptive statistics for demographics, anthropometrics, clinical and molecular characteristic were compared across subgroups of sensitivity/resistance to prior endocrine therapy and tested in uni/multivariate models. Clinical benefit was more common in sensitive patients with higher estrogen receptor expression and when fulvestrant was given in first line (*p*=0.02 and 0.046). In resistant patients, PFS was longer with lower BMI (*p*=0.01). Among endocrine sensitive women, longer PFS was associated with fulvestrant in first-line, single metastasis and no visceral involvement (*p*=0.01, 0.003 and 0.01). OS was shorter in resistant patients with HER2-positive disease and if fulvestrant was given in second and subsequent line (*p*=0.03). In sensitive patients, we observed worse OS with multiple metastases (*p*=0.008). Multivariate analyses confirmed longer PFS in resistant patients with lower BMI and older age (*p*=0.002 and 0.007). OS in resistant patients was negatively influenced by HER2 positivity and fulvestrant in second and subsequent line (*p*=0.04). In sensitive women, multiple metastases were associated with poorer survival (*p*=0.002). This evidence encourages considering patient and disease characteristics in decision making and outcome interpretation for patients candidate to fulvestrant.

## BACKGROUND

Endocrine therapy has become the mainstay of treatment for hormone receptor positive, human epidermal grown factor receptor 2 negative (HR+HER2-) breast cancer patients and often represents the first among several lines of treatment in metastatic disease. In this setting, the substantial expansion of the available armamentarium has undoubtedly translated into significantly improved outcomes [1].

Fulvestrant acts as a selective ER antagonist, which represses estrogen signaling throughout ER binding, conformational change and consequent block of dimerization followed by ER degradation and downregulation [2–3]. The clinical efficacy of fulvestrant was first shown in two phase III trials of fulvestrant 250 mg per month *versus* anastrozole 1 mg daily in 2^nd^ line [4–6]. Subsequently, consistent data in support of a dose-dependent effect of fulvestrant [7–9] prompted the design and conduct of the Comparison of Faslodex in Recurrent or Metastatic Breast Cancer (CONFIRM) trial, which showed improved progression-free survival (PFS) and overall survival (OS) in patients treated with a higher dose regimen including a day 14 loading element (500 mg on days 0, 14, and 28, and every 28 days thereafter) [10–11]. In the Fulvestrant First-Line Study Comparing Endocrine Treatments (FIRST), a phase II, randomized, open-label, multicenter trial fulvestrant 500-mg was compared with anastrozole in the 1^st^ line setting. A follow-up analysis reported a hazard ratio (HR) of time to progression (TTP) for fulvestrant 500 mg *versus* anastrozole of 0.66 (*p* = 0.01) [12]. Results from the overall survival analysis (OS) suggested that fulvestrant 500 mg extended OS compared to anastrozole (*p* = 0.04) [13]. These findings have recently found confirmation in the larger phase III FALCON (Fulvestrant and Anastrozole Compared in Hormonal Therapy Naïve Advanced Breast Cancer) trial (ClinicalTrials.gov identifier: NCT01602380), which has shown significantly longer PFS in the fulvestrant group than in the anastrozole group (*p* = 0.049) [14]. In its current use, fulvestrant is approved in postmenopausal HR+HER2- metastatic breast cancer following progression on previous endocrine treatment at a dose of 500 mg every 4 weeks, after an initial month of biweekly loading dose [15–16].

Endocrine resistance is a complex phenomenon whose roots have been increasingly investigated over the past decade. When addressing resistance to fulvestrant, with the lonely exception of ER, which is a widely accepted indicator of benefit from all endocrine therapies, the predictive role of additional factors have not been clarified yet. In these respects, a valid attempt was made by Graham and coauthors who carried out a systematic review and meta-analysis of 4 RCTs comparing fulvestrant based regimes to aromatase inhibitors (AI) alone. Time to progression/PFS was the primary endpoint. The authors observed longer TTP/PFS in patients treated with fulvestrant in case of visceral metastasis and time to recurrence greater than 5 years (*p* = 0.05 and *p* = 0.02, respectively) [17], while no relevant differences emerged when considering age and HER2 status (*p* = 0.32 and *p* = 0.09, respectively). However, results in patients with visceral site involvement may now be questionable in light of the results of the FALCON trial, which confirmed a substantial PFS advantage only in patients whose disease had not spread to liver or lungs (22.3 *vs* 13.8 months, in patients without and with visceral involvement, respectively) [14]. While discussing predictors of treatment outcomes in breast cancer patients undergoing endocrine therapies in the early setting, an interesting suggestion has recently come from a retrospective analysis of data from the transATAC (Arimidex, Tamoxifen Alone or Combined) trial. Results seem to encourage the incorporation of baseline demographic and anthropometric parameters, e.g., age and body mass index (BMI), into molecular scores for the prediction of distant recurrence in HR+HER2- breast cancer patients [18–19].

Given the scant and inconsistent evidence currently available on the potential determinants of treatment outcome in postmenopausal HR+ metastatic breast cancer treated with fulvestrant in 2^nd^ and subsequent line, we designed and conducted an observational study in a historic cohort of patients treated at several Italian cancer centers.

## RESULTS

Overall, the present analysis includes data for 161 patients, whose characteristics are shown in Table 1. In brief, medians and ranges for age, BMI, ER, PgR and Ki-67 percent expression were 68.9 years (35.0-87.0), 26.0 (16.2-45.3), 90 (1-100), 50 (1-100), and 15 (1-80), respectively. Most of our patients exhibited an ECOG PS equal to 0 [111 (68.9%)]. The HER2 receptor was overexpressed/amplified in 13 (8.1%) patients and metastatic involvement was revealed at one single site in 78 (48.4%) cases. Bones and viscera were exclusively involved by metastases in 59 (31.1%) and 17 (10.6%) women, respectively, while in the remaining cases (94, 58.3%) the metastatic spread followed a mixed pattern. Study participants characteristics across groups defined by endocrine resistance are shown in Table 2. The groups compared significantly differed in terms of BMI (*p* = 0.02), ER percentage expression (*p* = 0.03), HER2 status (0.04), and fulvestrant administration in 1^st^ line or subsequent line of therapy (*p* < 0.001). More specifically, in our cohort, endocrine resistant patients showed more commonly lower BMI, reached more rarely a 30% cut off for ER expression, were more commonly HER2+ and received more frequently fulvestrant in first line compared to endocrine sensitive patients.

**Table 1:** Descriptive characteristics of the study participants (N:161)

	Median	Range
**Age (years)**	68.9	35.0-87.0
**BMI (kg/m2)**	26	16.2-45.3
ER*	90	1-100
PgR*	50	1-100
Ki-67*	15	1-80
	**N**	**%**
**ECOG PS**		
**0**	111	68.9
***1***	37	23.0
***2***	7	4.3
***HER2***		
***positive***	13	8.1
***negative***	143	88.80
**Metastatic sites**		
***Number***		
***1***	78	48.4
***2***	65	40.4
***≥3***	18	11.2
***Pattern***		
***Bones***	50	31.1
***Viscera***	17	10.6
***Miscellanea***	94	58.4

*Reported values represent the medians and related ranges of ER/PgR receptor and Ki-67 percentage expression; BMI: body mass index; ECOG PS: Eastern Cooperative Oncology Group Performance Status; ER: estrogen receptor; PgR: progesterone receptor; HER2: human epidermal growth factor receptor 2.

**Table 2:** Descriptive characteristics of the study participants by endocrine Resistance/Sensitivity* (N:161)

Overall population	Endocrine-Resistant Patients 64 (39.75)	Endocrine-Responsive Patients 97 (36.6)	p
	**N(%)^§^**		
**Age** **<65** **≥65**	31(48.4) 33(51.6)	36(37.1) 52(53.6)	**0.36**
**ECOG PS** **0** **1-2**	49 (79.0) 13(21.0)	58(59.8) 27(27.8)	**0.15**
**BMI** **<*25*** **≥*25***	35(54.7) 29(45.3)	32 (33.0) 56 (57.7)	**0.02**
**ER 30** **<30** **≥30**	2(3.1) 61(95.3)	11(11.3) 67(69.1)	**0.03**
**PgR** **negative** **positive**	8(12.5) 56(87.5)	11(11.3) 77(79.4)	**1.0**
***HER2*** ***negative*** ***positive***	54(84.4) 9(14.1)	80(82.5) 4(4.1)	**0.04**
***Ki-67*** ***<15*** ***≥15*** ***Grade*** ***1-2*** ***3***	23(35.9) 38(59.4) 40(62.5) 18(28.1)	34(35.0) 43(44.3) 55(56.7) 22(22.7)	**0.45** **0.76**
**Fulvestrant given in** ***1st line*** ***2nd or subsequent*** ***Visceral Involvement*** ***no*** ***yes*** ***Number of metastatic sites*** ***1*** ***>1*** ***Bone involvement*** ***No*** ***Yes***	42(65.6) 22(34.4) 32(50.0) 32(50.0) 29(45.3) 35(54.7) 43(67.2) 21(32.8)	30(30.9) 58(59.8) 52(53.6) 36(37.1) 47(48.4) 41(42.3) 61(62.8) 27(27.8)	**<0.0001** **0.27** **0.32** **0.78**

*Patients were considered endocrine resistant if recurrence occurred during or within 12 months after the end of adjuvant treatment or progression was recorded while on or within 1 month after the completion of treatment for advanced disease.

§Percentages may not add up to 100 because of missing values

BMI: body mass index, ER and/or PgR: estrogen and/or progesterone receptor; ER: estrogen receptor; PgR: progesterone receptor.

None of the variables tested had a significant impact on OR, neither in endocrine sensitive nor in endocrine resistant patients (data available upon request). Conversely, within the subgroup of endocrine sensitive patients, Clinical benefit rate (CBR) was affected by percent expression of ER and line of therapy, with the highest rates being recorded in patients with a ER percentage expression of at least 30 and having received fulvestrant in 1^st^ line (*p* = 0.02 and 0.046, respectively) (data available upon request).

Results from univariate analyses addressing the role of anthropometric, clinical and molecular factors on survival across strata defined upon endocrine sensitivity/resistance are shown in Table 3.

**Table 3:** Univariate analysis of the impact of anthropometric, molecular and metabolic determinants on survival in endocrine sensitive (N:97) and endocrine resistant (N:64) patients HR+ metastatic breast cancer patients treated with fulvestrant

Variables	Median ^1^PFS (95% ^3^CI)				Median ^2^OS (95% CI)			
	Endocrine Sensitivity				Endocrine Sensitivity			
	No N: 64	p	Yes N: 97	p	No N: 64	p	Yes N: 97	p
**^4^BMI** **<25** **>=25**	7(6-7) 5(4-7)	0.01	7(5-9) 8(4-12)	0.10	35(15-55) 22(16-27)	0.36	23(2-44) 52(26-78)	0.05
**^5^HER2** **Pos** **Neg**	6(5-7) 5(3-6)	0.87	6 (0-17) 8(6-9)	0.60	12(7-17) 30(19-42)	0.03	n.r. 40(20-61)	0.32
**Age** **>=65** **<65**	6(4-7) 6(5-7)	0.40	8(7-10) 6(3-8)	0.05	24(12-35) 25(9-41)	0.97	n.a. 52(8-95)	0.09
**^6^ER%** **>=30** **<30**	6(5-7) n.a.	n.a.	8(6-9) 4 (3-5)	0.86	24(13-34) n.a.		40(25-55) n.a.	0.24
**^7^PgR** **Neg** **Pos**	6(0-13) 6 (5-8)	0.77	n.a. 8(6-9)	0.15	n.a. 24(12-35)	0.68	n.a. 41(23-59)	0.28
**Ki67%** **<15** **>=15**	6mesi (4-9) 6mesi (4-7)	0.73	7(5-8) 11(3-18)	0.074	21(17-26) 30(17-44)	0.41	41(19-63) n.a.	0.93
**Fulvestrant in 1st/subsequent line**: **1** **≥2**	7(5-8) 5(4-6)	0.09	10(6-15) 7(4-9)	0.01	32(19-44) 19(9-29)	0.03	n.r. 29(8-50)	0.17
**N metastatic sites** **1** **>1**	6 (5-7) 5(4-6)	0.85	10(2-18) 7(4-10)	0.003	35(22-49) 22(10-33)	0.11	n.a. 25(16-34)	0.008
**Visceral Involvement** **Yes** **No**	6 (5-8) 5 (3-7)	0.69	6(2-10) 9(3-15)	0.01	32(22-42) 19(8-30)	0.19	40(12-68) n.a.	0.09

In the endocrine resistant subgroup, PFS was significantly longer for patients in the lowest category of BMI (*p* = 0.01), while, among endocrine sensitive women, longer PFS was observed in cases treated with fulvestrant in 1^st^ line (*p* = 0.01), with metastatic involvement limited to one single site (*p* = 0.01) and with no extension to visceral sites (*p* = 0.003). Among endocrine resistant patients, HER2 positive status was associated with poorer OS (*p* = 0.03), similarly to having received fulvestrant in 2^nd^ and subsequent lines (*p* = 0.03). In endocrine sensitive patients, we observed worse outcome in case of multiple metastatic sites (*p* = 0.008). In multivariate analysis (Table 4), for endocrine resistant patients, lower BMI and older age appeared associated with lower risk of progression (*p* = 0.02 and 0.007, respectively), while in endocrine sensitive patients we observed some evidence suggesting longer PFS in women who were older at diagnosis and had only one site involved by metastatic spread (*p* = 0.06 for both). When addressing OS, in endocrine resistant patients, HER2 positivity conferred a more than two-fold risk, along with fulvestrant administration in second and subsequent line of therapy (*p* = 0.04 for both). In endocrine sensitive women, the involvement of multiple metastatic sites was strongly associated with worse outcome (*p* = 0.002).

**Figure 1:**
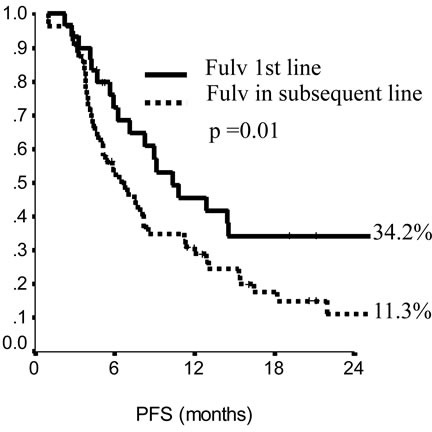
Progression free survival (PFS) in endocrine sensitive patients The impact of line of therapy (fulvestrant in first versus subsequent line).

**Table 4:** Multivariate analysis of the impact of anthropometric, molecular and metabolic determinants on survival in endocrine sensitive (N:97) and endocrine resistant (N:64) patients HR+ metastatic breast cancer patients treated with fulvestrant

Variables	HR (95% CI) PFS				HR OS (95% CI)			
	Endocrine Sensitivity				Endocrine Sensitivity			
	No N: 64	P	Yes N:97	p	No N: 64	p	Yes N:97	p
**BMI** >=25 vs <25	1.89 (1.11-3.24)	0.02	-	-	-	-	1.89(0.94-3.82)	0.08
HER2 **Pos vs Neg**	-	-	-	-	2.48(1.04-5.91)	0.04	-	-
**Age**	0.97(0.94-0.99)	0.007	0.98(0.95-1.0)	0.06	-	-	-	-
**Fulvestrant in subsequent line vs 1st**	-	-	1.89(1.08-3.31)	0.03	2.13(1.04-4.33)	0.04	-	-
**N metastatic sites** **>1 vs 1**	-	-	1.69(0.97-2.93)	0.06	-	-	3.32(1.56-7.13)	0.002
**Visceral involvement** **Yes vs No**	-	-	1.58(0.92-2.72)	.10	-	-	-	-

## DISCUSSION

Within the study herein presented, we analyzed data of 161 postmenopausal HR+ metastatic breast cancer patients treated with fulvestrant in 1^st^ and subsequent line of therapy following development of resistance to AI. Based on the outcome related to this latter treatment, patients were defined as endocrine sensitive or resistant. The overall analytical approach was thus based on the stratification by response to prior endocrine therapy. This allowed to highlight how distinct determinants may affect treatment efficacy within these two groups of postmenopausal HR+ metastatic breast cancer patients treated with fulvestrant. In brief, CBR was more commonly achieved in endocrine sensitive patients with higher ER percent expression and in those who received fulvestrant in 1^st^ line, while no specific determinants seemed to affect CBR at a significant extent in endocrine resistant patients. Progression free survival appeared to be positively influenced by lower BMI in endocrine resistant patients, whereas treatment administration in 1^st^ line, one single metastatic site and no visceral involvement were associated with sensibly longer PFS in endocrine sensitive patients. Univariate analysis of OS tested significant for HER2 status and line of treatment in endocrine resistant patients, who were disfavored in case HER2 positivity and 2^nd^ or subsequent line fulvestrant, while endocrine sensitive patients showed worse outcomes in case of multiple metastatic involvement. As expected, multivariate models confirmed a restricted number of factors. More specifically, PSF resulted longer in resistant patients who were older at diagnosis and showed lower BMI, while, in endocrine sensitive patients there was some evidence in support of more favorable outcomes related with older age and single metastatic site involvement. Overall survival was shorter in endocrine resistant patients expressing HER2 and having been treated with fulvestrant in 2^nd^ and subsequent lines. In endocrine sensitive patients, worse OS was associated with multiple metastatic site involvement.

**Figure 2:**
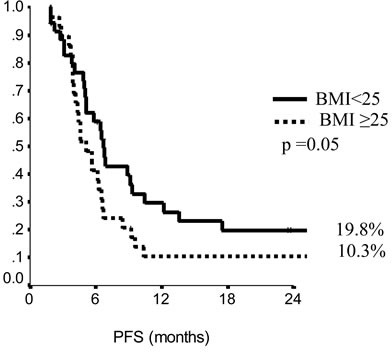
Progression free survival in endocrine resistant patients

**Figure 3:**
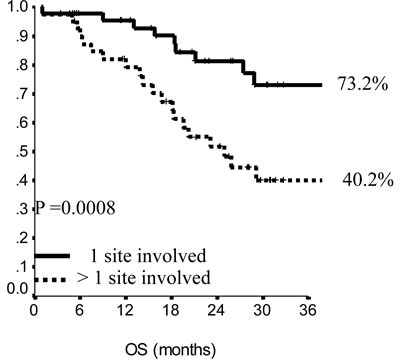
Overall survival in endocrine sensitive patients

The analytic approach used by our group represents a shortcut to the immediate use of data from the real world population setting in orienting towards the most suitable therapeutic approach at the single patient level based on the outcome of prior exposure to endocrine therapy. Such a feasible approach is not meant to be exhaustive in providing all the key elements required for the most appropriate therapeutic choice within the rapidly expanding armamentarium of endocrine agents, but to add informative pieces to the overall puzzle based on promptly available patient- and disease-related info. This may result particularly attractive in light of some criticisms emerging from the a rapid overview of the most recent literature on fulvestrant in HR+ locally advanced and metastatic breast cancer. As previously mentioned, the FALCON trial was carried out in endocrine naïve patients [14]. This has made its results fairly comparable to those from the FIRST trial, whose participants had be mostly not exposed to endocrine therapy prior to study entry. Results from both these studies have undoubtedly concurred to provide evidence concerning superior efficacy of fulvestrant over 3^rd^ generation aromatase inhibitors. Yet, given the characteristics of the overall population enrolled in the phase III study and in the FIRST trial, this evidence is barely generalizable to patients from the real world setting and, as such, not comparable to our study results. When looking beyond recent literature from randomized clinical trials, fulvestrant use in HR+ advanced breast cancer has been addressed by Gevorgyan and colleagues, who examined the role of BMI on CBR in a retrospective study of seventy-five consecutive patients treated with fulvestrant at a single institution. The authors observed better outcomes in normal-weight patients compared with underweight and overweight/obese patients (CBR: 70.0, 31.6 and 25.0%, respectively, *p* < 0.001) [20]. In our study population, we observed no effects of BMI on CBR. However, our results support a role of BMI on PFS, with longer PFS being observed in endocrine resistant patients with lower BMI values. This latter evidence surely represents the most intriguing result from our analysis. Indeed, with the lonely exception of the study from Gevorgyan and colleagues [20], no other study has previously addressed the predictive role of BMI on treatment outcome in breast cancer patients treated with fulvestrant. More generally, there is paucity of data concerning the influence of BMI on endocrine therapy outcome in the advanced setting. According to the results reported by Michaud et al., BMI had no predictive value on TTP and response rates in metastatic breast cancer patients treated with anastrozole compared with tamoxifen. Similar conclusions were drawn by Schmid and coauthors when analyzing data from patients treated with letrozole or megestrol acetate [21–23]. Conversely, evidence from our study seems to encourage the use of fulvestrant in patients with lower BMI who showed prior hormone resistance. Within the limits imposed by the relatively restricted sample size and observational nature of our study, we may speculate that both estrogen- and non-estrogen dependant mechanisms may concur to explain the outcome observed. In patients with higher BMI, a greater volume of adipose tissue may be associated with an overall increased aromatase activity. It is also plausible that in obese and overweight patients higher insulin levels may activate fetal insulin/IGF-1 receptors on breast cancer cells and activate cell signaling through PI3K and Ras-Raf pathways. In addition, the cross-talk between IGF-1/insulin signaling pathways and estrogenic signaling pathways is well documented. Thus, enhanced signaling mediated by the insulin IGF-1 pathway in overweight and obese patients may activate estrogen signaling pathways [24–26].

**Figure 4:**
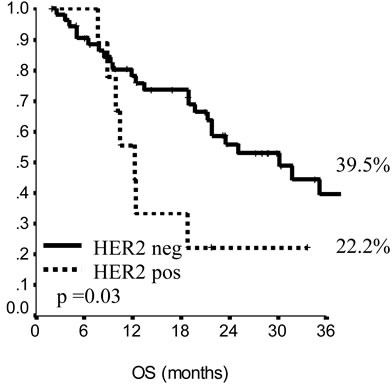
Overall survival in endocrine sensitive patients

**Figure 5:**
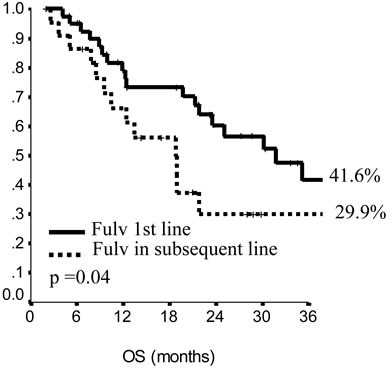
Overall survival in endocrine sensitive patients

To our knowledge, no further evidence potentially useful to inform decisions on fulvestrant use in advanced breast cancer has recently come from the clinical setting. Less recent work has been carried out by Knudsen and colleagues on the development and validation of a gene expression score to predict response to fulvestrant in the neoadjuvant setting. The potential predictor was applied to tissue specimen sampled before neoadjuvant fulvestrant in patients from a phase II trial. The inclusion of clinical covariate, tumor grade and ER expression increased the difference in sensitivity between responders and not responders (*p* = 0.003) [27]. Beyond its limitations, punctually addressed by the authors in the discussion, results from this study seem to be particularly encouraging concerning the combined use of new-fashion techniques and old-fashion approaches, i.e., gene signatures coupled with tumor- and patient-features possibly including BMI, in the decision making process eventually leading to fulvestrant administration. This strategy may result effective across different breast cancer setting, In specific regard to the advanced setting for postmenopausal HR+HER2-breast cancer, such an approach may result of particular interest in re-defining the therapeutic continuum for candidates to endocrine therapy following the FDA approval of the cycline dependent inhibitor palbociclib in combination with endocrine agent [28].

Our study has some limitations, mostly related to its design and placement within the real world clinical setting. Clinical series are by nature prone to confounding and bias, which should always invite caution in results’ interpretation. Missing values for the variables of interest were highlighted when presenting results in the pertinent tables. Unfortunately, systematic data collection and update in course of follow up have not stably entered the clinical practice settings yet, and the related processes are still amenable to implementation. The multicentric design has greatly improved our enrollment abilities, but no central assessment has been performed on the samples from included participants. However, the dedicated labs at the enrolling institutions are constantly involved in quality controls and ISO 9001-certied.

Our study also have strengths of relevance. In first place, our study provides evidence in support of differentials patient- and disease-related characteristics related to response to prior exposure to endocrine therapy. At the same time, this may add in terms of interpretations of the outcomes from a previous treatment and inform future decisions within an increasingly articulated therapeutic strategy which is not limited to the immediately upcoming therapeutic choice. This may translate into a more appropriate short- and mid-term therapeutic planning for our patients. In addition, data from a non selected population, which does not satisfy strict inclusion and exclusion criteria to allow inclusion in randomized clinical trial, are more easily adaptable to the needs of both real world patients and actively practicing oncologists.

In brief, we carried out an analysis of data from 161 postmenopausal HR+ metastatic breast cancer patients who had received fulvestrant in 1^st^ and subsequent line of therapy following development of resistance to AI. Stratification by endocrine sensitivity/resistance helped identify factors significantly differing across the groups compared. Among such differential traits, several determinants had a relevant impact on patients’ important outcomes. Pending our results’ confirmation in future studies, such an approach may be efficiently integrated by the results of genomics and transcriptomics techniques to more appropriately inform fulvestrant use, and, more generally, therapeutic decisions in HR+ metastatic breast cancer.

## MATERIALS AND METHODS

The analysis herein proposed includes data from a larger study of postmenopausal HR+ metastatic breast cancer patients who had developed resistance following endocrine therapy with AI. Details on methods have been extensively provided elsewhere [29]. In brief, in this cohort, women were categorized based on their response in terms of resistance/responsiveness to prior endocrine therapy. In more details, patients previously exposed to endocrine therapy in the adjuvant setting were judged “hormone resistant” if their disease recurred while on treatment or within 12 months after the end of adjuvant therapy. Similarly, those patients who had received prior endocrine therapy in the advanced setting were considered “hormone resistant” if disease progression occurred while on treatment or within 1 month after the completion of therapy. Prior chemotherapy regimens and anticancer endocrine therapies for advanced disease were allowed. An Eastern Cooperative Oncology Group (ECOG) performance status (PS) of 2 or less and adequate organ and hematologic functions were requested. Asymptomatic CNS metastases were not an exclusion criterion. Surgery or radiation therapy of brain lesions was admitted if performed within 3 months preceding study entry. Bisphosphonate treatment was allowed. Fulvestrant was administered according to the current indications and recommendations [15–16].

Five Italian cancer centers contributed data to the present analysis. An ad hoc database was conceived and implemented for data collection. Data of interest were pertinent to patient demographics, anthropometrics (weight, eight, BMI), clinical and morphological features, treatment/s administered and related outcomes. When considering ER percent expression in both descriptive and inferential statistics, we set up two different cut off levels, i.e., 30 and 50%. The related categorical variables were both tested, with results being reported according to the most conservative choice, i.e., a cut off value of 30%, unless the two cut off values generated significantly differences. To the purpose of our analysis, BMI was computed as weight in kilograms divided by the square of the height in meters and addressed as a categorical variable using a 25 cut off value. Among the outcomes of interest, objective response (OR) was codified according to conventional Response Evaluation Criteria in Solid Tumours (RECIST) version 1.1. Clinical benefit rate CBR was addressed as the percentage of patients with shrinking tumors or stable disease for at least 6 months. Progression free survival was defined as the time elapsed between treatment start and interruption due to disease progression or death from any cause. Overall survival was defined as the time from the start of treatment to patient death from any cause. The study was conducted in accordance with Helsinki Declaration and approved by Independent Ethical Committees of the institutes involved. All the patients provided their written informed consent.

### Statistical analysis

Descriptive characteristics were first approached for the overall study population and for all the variables of interest. Medians and ranges were computed for continuous variables, while frequencies and percentages were used for categorical variables. Patients’ characteristics were then compared across groups defined upon endocrine resistance in full accordance with the above reported definition using X^2^/Fisher test. Any statistically relevant difference emerging from this latter comparison was further considered in analyses testing the impact of anthropometric, clinical and molecular features on treatment outcomes. The Hazard Ratio and confidence limits (CI) were estimated for each variable using the Cox univariate model. Significance was defined at the *p*≤0.05 level. A multivariate Cox hazard model was developed using stepwise regression (forward selection) by selecting significant variables upon univariate analysis. Enter limit and remove limit were *p* = 0.05 and *p* = 0.10, respectively. Survival curves were calculated by the Kaplan-Meier method and the log-rank test was used to assess differences between subgroups. Significance was defined at the *p*≤0.05 level. Statistical analyses were performed with SPSS statistical software version 20 (SPSS inc., Chicago IL, USA).
